# Drought-hardening improves drought tolerance in *Nicotiana tabacum* at physiological, biochemical, and molecular levels

**DOI:** 10.1186/s12870-020-02688-7

**Published:** 2020-10-23

**Authors:** Rayyan Khan, Xinghua Ma, Shahen Shah, Xiaoying Wu, Aaqib Shaheen, Lixia Xiao, Yuanhua Wu, Shusheng Wang

**Affiliations:** 1 Tobacco Research Institute, Chinese Academy of Agricultural Sciences, Key Laboratory of Tobacco Biology and Processing, Ministry of Agriculture, Qingdao, 266101 China; 2 Graduate School of Chinese Academy of Agricultural Sciences, Beijing, 100081 China; 3 Department of Agronomy, The University of Agriculture Peshawar, Peshawar, 25130 Pakistan; 4 Key Laboratory of Plant Stress Biology, State Key Laboratory of Crop Stress Adaptation and Improvement, School of Life Sciences, Henan University, Kaifeng, 475004 China

**Keywords:** Tobacco, *Nicotiana tabacum*, Drought-hardening, Drought tolerance, Varieties, Antioxidant enzymes, Gene expression, *SnRK2*, *AREB*, *DREB*

## Abstract

**Background:**

Drought stress is the most harmful one among other abiotic stresses with negative impacts on crop growth and development. Drought-hardening is a feasible and widely used method in tobacco seedlings cultivation. It has gained extensive interests due to its role in improving drought tolerance. This research aimed to investigate the role of drought-hardening and to unravel the multiple mechanisms underlying tobacco drought tolerance and adaptation.

**Results:**

This study was designed in which various drought-hardening treatments (CK (no drought-hardening), T1 (drought-hardening for 24 h), T2 (drought-hardening for 48 h), and T3 (drought-hardening for 72 h)) were applied to two tobacco varieties namely HongHuaDaJinYuan (H) and Yun Yan-100 (Y). The findings presented a complete framework of drought-hardening effect at physiological, biochemical, and gene expression levels of the two tobacco varieties under drought stress. The results showed that T2 and T3 significantly reduced the growth of the two varieties under drought stress. Similarly, among the various drought-hardening treatments, T3 improved both the enzymatic (POD, CAT, APX) and non-enzymatic (AsA) defense systems along with the elevated levels of proline and soluble sugar to mitigate the negative effects of oxidative damage and bringing osmoregulation in tobacco plants. Finally, the various drought-hardening treatments (T1, T2, and T3) showed differential regulation of genes expressed in the two varieties, while, particularly T3 drought-hardening treatment-induced drought tolerance via the expression of various stress-responsive genes by triggering the biosynthesis pathways of proline (*P5CS1*), polyamines (*ADC2*), ABA-dependent (*SnRK2*, *AREB1*), and independent pathways (*DREB2B*), and antioxidant defense-related genes (*CAT*, *APX1*, *GR2*) in response to drought stress.

**Conclusions:**

Drought-hardening made significant contributions to drought tolerance and adaptation in two tobacco variety seedlings by reducing its growth and, on the other hand, by activating various defense mechanisms at biochemical and molecular levels. The findings of the study pointed out that drought-hardening is a fruitful strategy for conferring drought tolerance and adaptations in tobacco. It will be served as a useful method in the future to understand the drought tolerance and adaptation mechanisms of other plant species.

**Graphical abstract:**

Drought-hardening improved drought tolerance and adaptation of the two tobacco varieties. T1 indicates drought-hardening for 24 h, T2 indicates drought-hardening for 48 h, T3 indicates drought-hardening for 72 h 
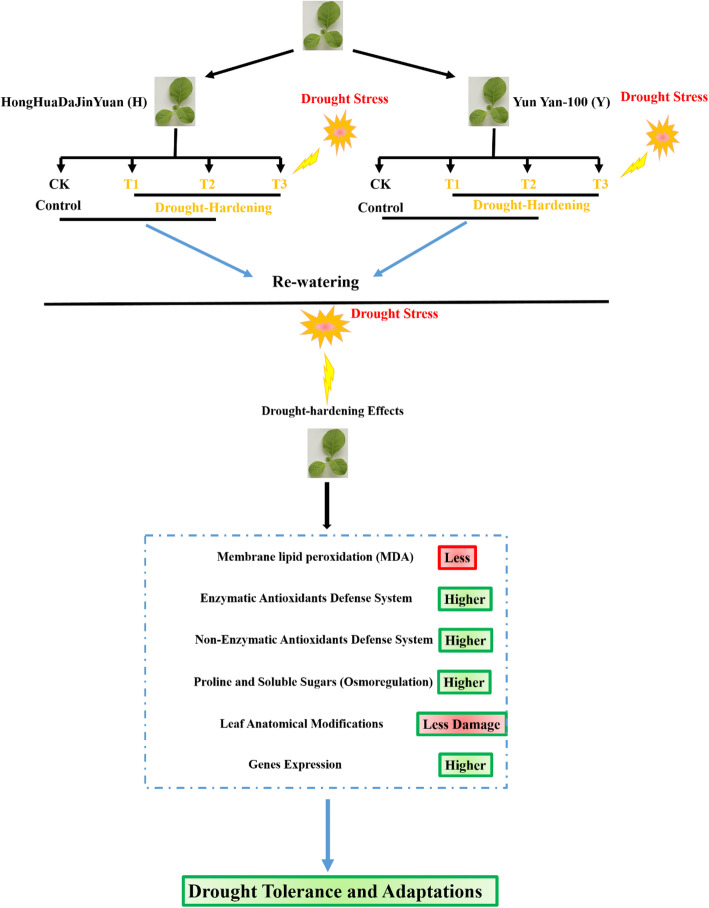

## Background

Agriculture and climate change are interrelated to each other, and the global climate is changing more rapidly than ever before [1]. Water limitation is a major constraint in global crop growth, development, and production, and climate change is the leading cause of it, thus worsening this situation extravagantly with adverse effects on agriculture in most regions of the world [2, 3]. Drought is one of the most important abiotic stresses, which causes negative effects on plant growth and productivity due to physical damage, physiological and biochemical disruptions, and molecular changes, which results in abnormal metabolism, reduced growth, and ultimately plant death [4–6]. The reduction in plant growth and development depends upon the severity and duration of drought stress. To cope with and overcome the drought stress, plants evolve several strategies, defense and adaptation mechanisms, comprised of morphological, physiological, biochemical, and molecular modifications [7]. The abovementioned modifications related to defense and adaptation to drought stress were stomata closure, cellular adaptations, membrane stability, carbon fixation rate, reactive oxygen species scavenging system, hormonal regulation, induction of stress-related genes, signaling genes, and stress proteins. These modifications have been found in playing a crucial role in plant survival under drought stress [8, 9].

The effect of drought stress on plants can be minimized and provide tolerance through the application and manipulation of various methods/techniques. These methods/techniques and adapting strategies which provide drought tolerance in plants are breeding techniques, exogenous application of phytohormones and osmoprotectants to plants or seeds, and drought-hardening [10, 11]. The young seedlings are malleable so their drought tolerance can be improved via their exposure to earlier low water stress. Therefore, drought-hardening is a feasible and convenient method that involves the utilization of reduced or partial irrigation of seedlings to pre-condition the seedlings to the drought stress [12]. Drought-hardening neither improves the drought tolerance but also provides tolerance in plants to other abiotic stresses like cold stress in tomato [13], chilling stress in cucumber seedlings [14], and also provides cross adaptation to heavy metals in spring barley [15]. Several studies had proven the importance of drought-hardening in various plant species that help in mitigating the negative impacts of drought stress and provide drought tolerance to plants [11, 16].

Drought-hardening improved the drought tolerance of plants through morphological manifestations (improved root/shoot ratio, leaf extension, root surface area) [16–18], physiological adaptations (transpiration rate, photosynthesis rate, relative water content, osmotic potential, chlorophyll content) [17, 19], biochemical adaptations (improved antioxidant defense system, proline content, soluble sugars, polyamines) [19], and molecular manifestations with the higher expression of genes related to proline and glycine betaine pathways in drought- hardened plants than that in non-drought hardened plants under drought stress [20]. The survival of the seedlings could be improved by its exposure to different durations and intensities of water stress before transplantation. Various studies showed the different duration of time intervals for drought-hardening. The water stress time duration for drought-hardening ranges from a few days [20, 21] to weeks [19, 22] in various plant species. In our previous study Khan et al. [23], drought stress for 2 or 3 days exhibited deleterious effects on plants which showed yellowing and wilting of leaves. On this basis, that time point durations were used for drought-hardening in this study.

Drought stress led to accumulation of reactive oxygen species (ROS) in a higher amount which is detrimental to plant growth. To mitigate the effect of ROS, plants induce higher antioxidant enzyme activities, and higher expression of their related genes [24, 25], which helps in conferring drought stress tolerance and adaptation. Besides, from the regulation of antioxidant enzymes activities and their related genes expression in response to drought stress, various other genes were also regulated and highly expressed such as osmolytes biosynthesis-related genes (*P5CS*, *ADC2*) [26], signaling gene like *SnRK2* [27], transcription factors like *WRKY* [28, 29], *AREB* and *DREB* [30], thus conferring drought stress tolerance and adaptation in plants. These genes could be utilized for highlighting the role of drought-hardening in conferring drought tolerance in tobacco.

Various researches pointed out the importance of drought-hardening in providing drought tolerance in several crops. In this study, we investigated two tobacco variety seedlings at the physiological, biochemical, and molecular levels to gain a more comprehensive understanding of the mechanisms of providing drought tolerance via drought-hardening under drought stress.

## Results

### Changes in growth, chlorophyll fluorescence, and multicolor fluorescence parameters

**Fig. 1:**
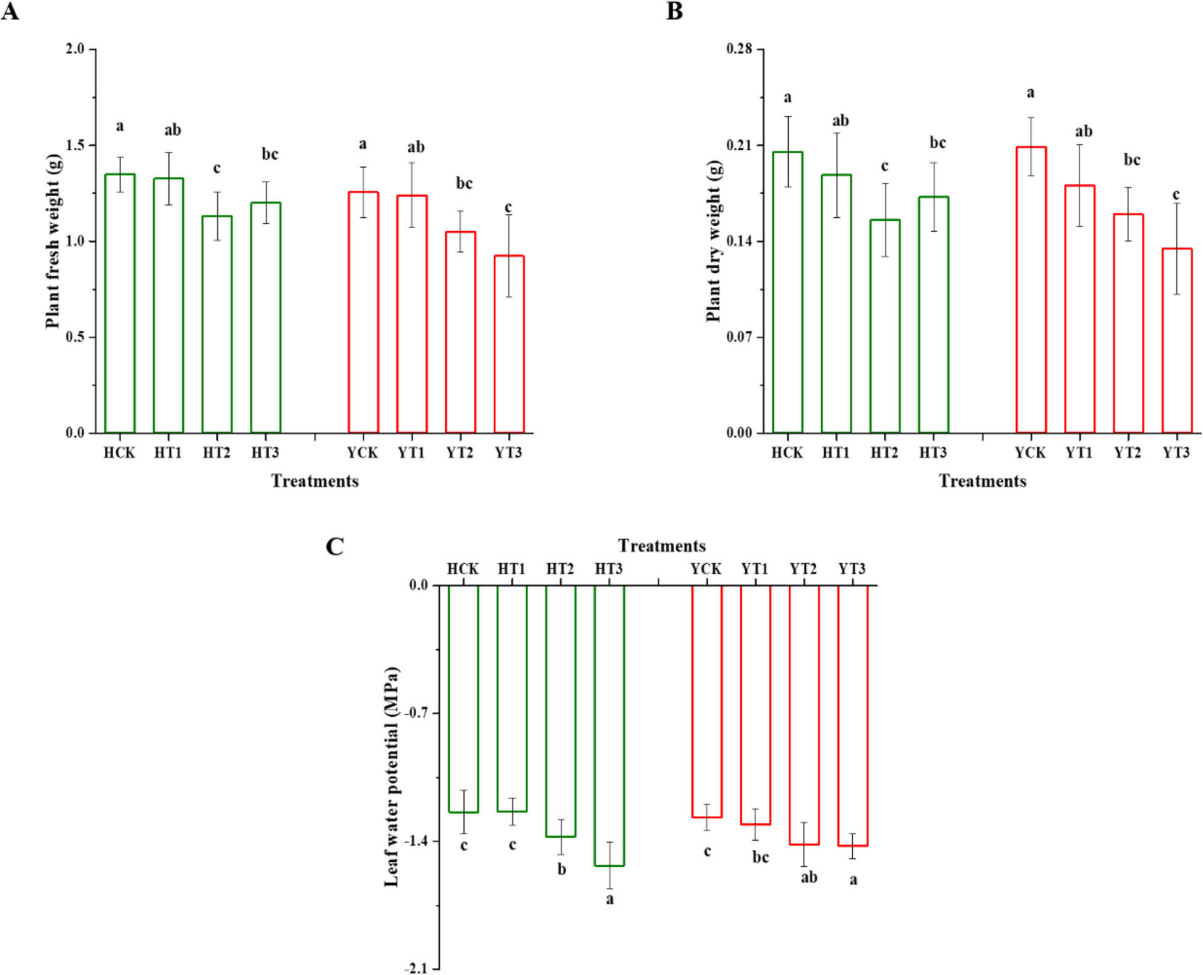
Influence of drought-hardening on growth indices and leaf water potential (LWP) of two tobacco varieties in response to drought stress. Plant fresh weight (**a**), plant dry weight (**b**), and LWP (**c**) of the various drought-hardening and non-drought-hardening treatments under drought stress. The values are presented as means ± SDs. Different letters indicate a significant difference using the least significant difference (LSD) test, *p* < 0.05. HCK presents HongHuaDaJinYuan seedlings with no drought-hardening; HT1 indicates 24 h drought-hardened seedlings of HongHuaDaJinYuan; HT2 indicates 48 h drought-hardened seedlings of HongHuaDaJinYuan; HT3 indicates 72 h drought-hardened seedlings of HongHuaDaJinYuan; YCK represents Yun Yan-100 seedlings with no drought-hardening; YT1 indicates 24 h drought-hardened seedlings of Yun Yan-100; YT2 indicates 48 h drought-hardened seedlings of Yun Yan-100; YT3 indicates 72 h drought-hardened seedlings of Yun Yan-100

The drought tolerance of the two tobacco cultivars was analyzed under drought stress by applying various drought-hardening treatments. Significant differences were observed in the plant fresh (PFW) and dry weight (PDW) of the two cultivars in various drought-hardening treatments by applying drought stress (Fig. 1a and b). The PFW and PDW of H were reduced by 16 and 11%, 24 and 16% for T2 and T3, and PFW and PDW of Y was reduced by 16 and 26%, and 24 and 36% for T2 and T3, respectively, in response to drought stress. Similarly, the leaf water potential (LWP) was also monitored in drought-hardened and control plants of the two tobacco varieties under drought stress. There was a significantly pronounced decrease observed in T2 and T3 treatments of both varieties, in contrast, to control, while T1 and control were statistically similar in response to drought stress (Fig. 1c). The decrease for HT2 was 11 and 10% for YT2, while 24% for HT3 and 12% for YT3 in response to drought stress in comparison to their respective controls.

**Fig. 2:**
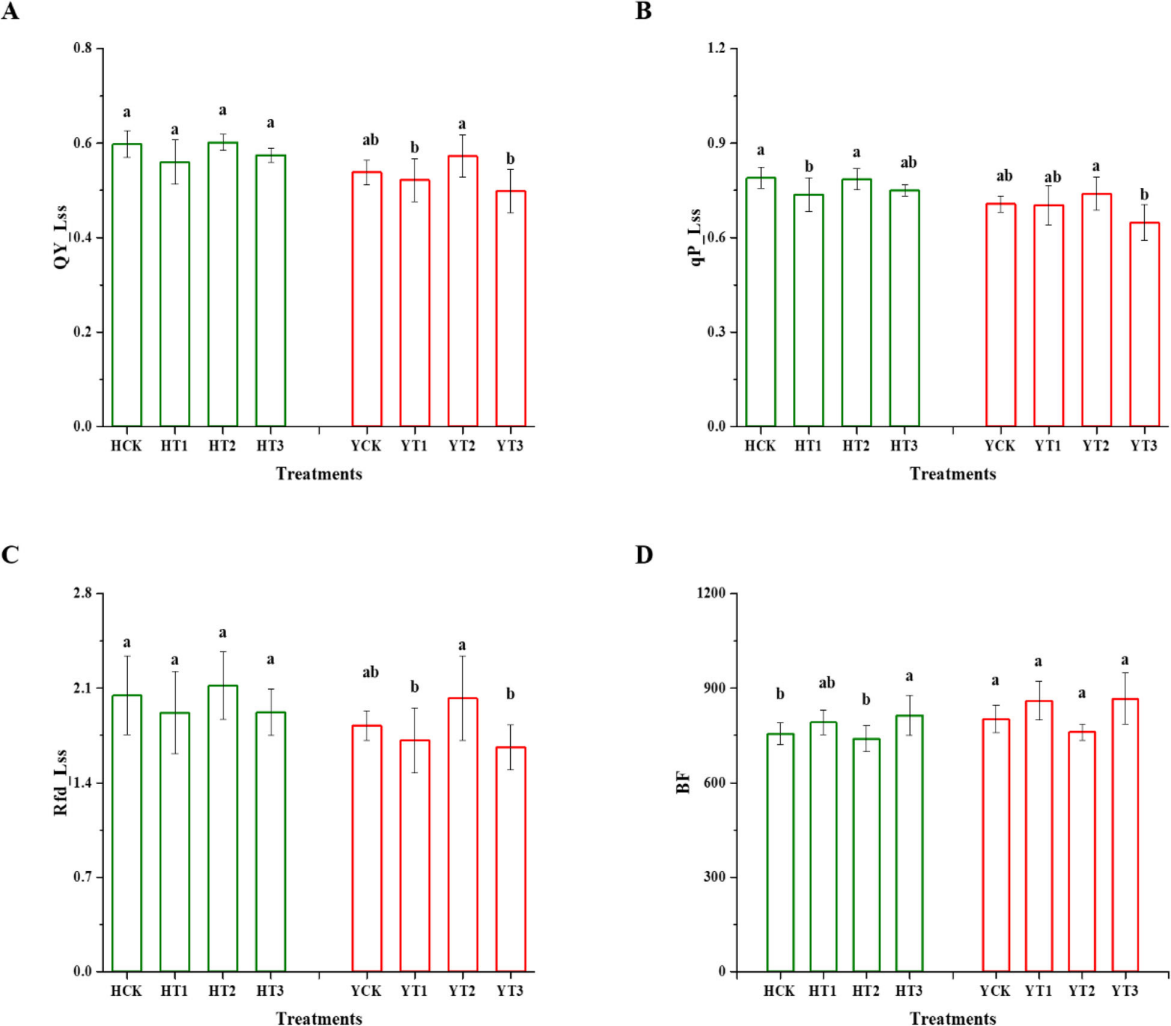
Effect of drought-hardening on chlorophyll and multicolor fluorescence parameters in response to drought stress. PSII quantum Yield (QY_Lss) (**a**), open reaction centers (qP_Lss) (**b**), a ratio of fluorescence decline (Rfd_Lss) (**c**), and blue fluorescence (BF) (**d**) of the various drought-hardening and non-drought-hardening treatments. Data represented means ± SD. Different letters indicate a significant difference at LSD, *p* < 0.05. HCK represents HongHuaDaJinYuan seedlings with no drought-hardening; HT1 indicates 24 h drought-hardened seedlings of HongHuaDaJinYuan; HT2 indicates 48 h drought-hardened seedlings of HongHuaDaJinYuan; HT3 indicates 72 h drought-hardened seedlings of HongHuaDaJinYuan; YCK represents Yun Yan-100 seedlings with no drought-hardening; YT1 indicates 24 h drought-hardened seedlings of Yun Yan-100; YT2 indicates 48 h drought-hardened seedlings of Yun Yan-100; YT3 indicates 72 h drought-hardened seedlings of Yun Yan-100

Figure 2 represented the chlorophyll fluorescence parameters, which determine the photosynthetic performance of the drought-hardened and non-drought-hardened plants of the two tobacco varieties under drought stress. In both cultivars, qP_Lss (open reaction centers) declined significantly in drought-hardened plants in T1 of H with a 7% reduction in comparison with non-drought-hardened plants, while an 8% reduction was observed for T3 in Y (Fig. 2b). Furthermore; there were significant changes observed in QY_Lss and Rfd_Lss in T1 and T3 of Y with a 3 and 7%, 6, and 9% reduction, respectively, in comparison with non-drought-hardened plants (Fig. 2a and c). Blue fluorescence (BF) is a multicolor fluorescence (MCF) parameter which was selected from basic MCF parameters. It was observed that drought-hardening, especially T3 showed a significant rise in BF under drought stress in H seedlings in comparison with control, while drought-hardening had no significant effect on BF in Y seedlings (Fig. 2d).

**Fig. 3:**
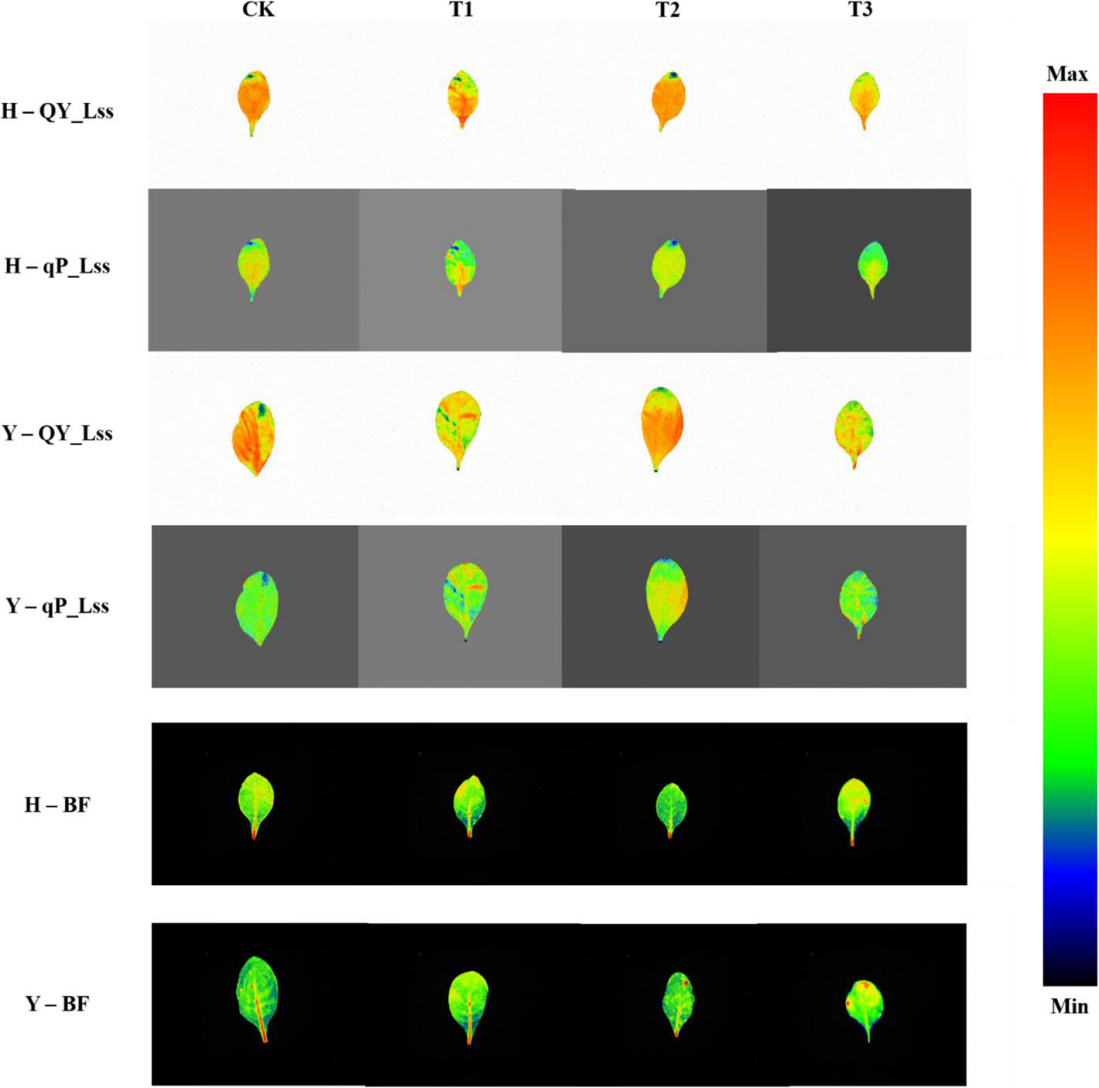
Representative chlorophyll and multicolor fluorescence images. PSII quantum yield (QY_Lss) and qP_Lss (open reaction centers) and multicolor fluorescence images of blue fluorescence (BF) of the two tobacco varieties, HongHuaDaJinYuan (H) and Yun Yan-100 (Y) of various drought-hardening treatments along with control under drought stress. CK represents no drought-hardening; T1 indicates drought-hardening for 24 h; T2 indicates drought-hardening for 48 h; T3 indicates drought-hardening for 72 h

The representative images of QY_Lss and qP_Lss and one parameter of MCF, BF of control, and drought-hardened plants of the two varieties under drought stress were shown in Fig. 3. The images of QY_Lss and qP_Lss of both varieties showed variation, and its signal intensity was decreased from the tips and margins to the entire leaf showing spatial heterogeneity, especially in T3. Similarly, the intensity of BF signals was increased from the middle to edges and tips of the leaves of T3, also exhibiting spatial variation.

### Changes in malondialdehyde (MDA) and hydrogen peroxide (H_2_O_2_)

**Fig. 4:**
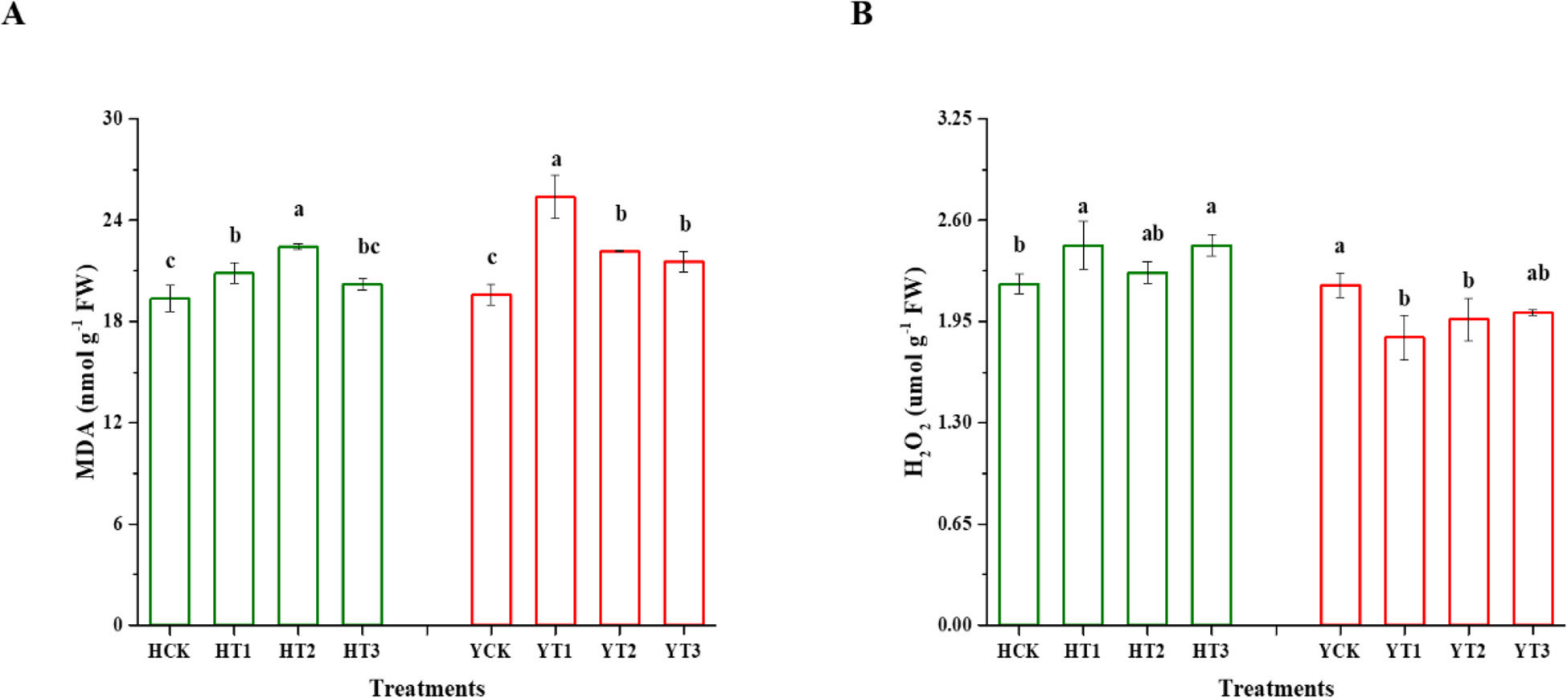
Oxidative stress response of drought-hardened tobacco seedlings against drought stress. The contents of MDA (**a**) and H_2_O_2_ (**b**). Drought stress was applied to the plants of various drought-hardening treatments along with control. Data are the means ± SDs (different letters indicate significant differences at *p* < 0.05 by LSD test). HCK represents HongHuaDaJinYuan seedlings with no drought-hardening; HT1 indicates 24 h drought-hardened seedlings of HongHuaDaJinYuan; HT2 indicates 48 h drought-hardened seedlings of HongHuaDaJinYuan; HT3 indicates 72 h drought-hardened seedlings of HongHuaDaJinYuan; YCK represents Yun Yan-100 seedlings with no drought-hardening; YT1 indicates 24 h drought-hardened seedlings of Yun Yan-100; YT2 indicates 48 h drought-hardened seedlings of Yun Yan-100; YT3 indicates 72 h drought-hardened seedlings of Yun Yan-100

Figure 4 showed changes in the MDA and H_2_O_2_ contents in the drought-hardened and non-drought hardened plants of H and Y cultivars in response to drought stress. There were significant differences observed between the various treatments compared with control in both varieties. HT3 treated plants and control plants are statistically similar, while 8 and 16% increase was witnessed in the MDA content of HT1 and HT2, respectively, when plants were exposed to drought stress. Similarly, drought stress culminated 30, 13, and 10% rise of the MDA content in YT1, YT2, and YT3 plants, respectively, when compared with the YCK. As can be seen in Fig. 4b, the same increase of 11% was observed in the H_2_O_2_ content of HT1 and HT3. Similarly, 15 and 10% decline occurred in the H_2_O_2_ content of YT1 and YT2 upon the imposition of drought stress, respectively.

### Changes in antioxidant enzyme activities

**Fig. 5:**
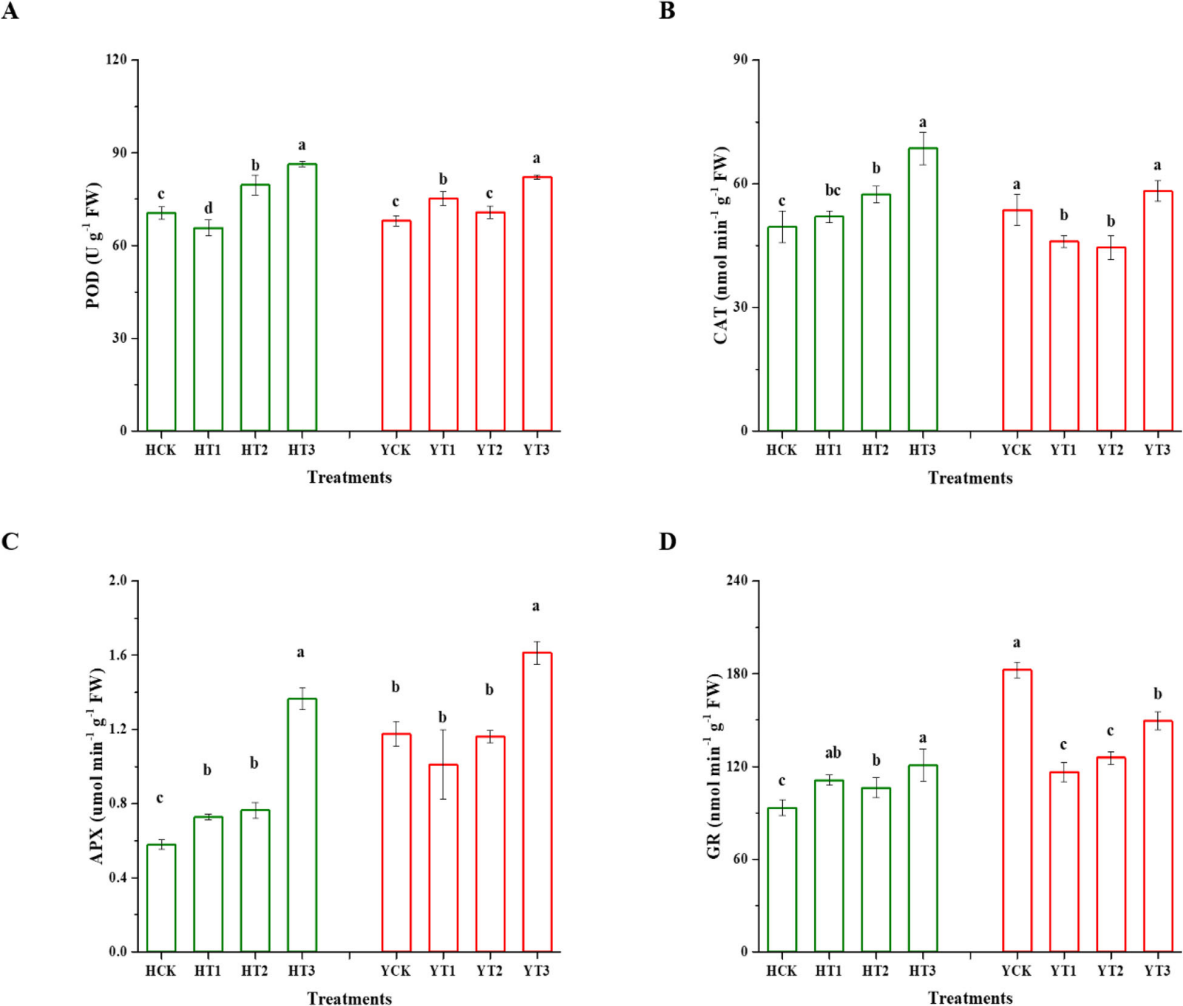
Effect of drought-hardening on the antioxidant enzyme activities in response to drought stress. Drought stress-induced alterations in the antioxidant enzyme activities, POD (**a**), CAT (**b**) APX (**c**), and GR (**d**), of the two tobacco variety of various drought-hardening treatments seedlings along with non-drought-hardened seedlings. Means presented with error bars are ± SDs, different letters indicating significant differences at *p* < 0.05 by LSD test. HCK represents HongHuaDaJinYuan seedlings with no drought-hardening; HT1 indicates 24 h drought-hardened seedlings of HongHuaDaJinYuan; HT2 indicates 48 h drought-hardened seedlings of HongHuaDaJinYuan; HT3 indicates 72 h drought-hardened seedlings of HongHuaDaJinYuan; YCK represents Yun Yan-100 seedlings with no drought-hardening; YT1 indicates 24 h drought-hardened seedlings of Yun Yan-100; YT2 indicates 48 h drought-hardened seedlings of Yun Yan-100; YT3 indicates 72 h drought-hardened seedlings of Yun Yan-100

The activities of the antioxidant enzyme (POD, CAT, APX, and GR) were monitored in H and Y seedlings and significantly affected (*p* < 0.05) in various drought-hardening treatments under drought stress (Fig. 5). POD activity was improved by 13% in HT2 and 22% in HT3 in comparison with control (HCK) under drought stress. Similarly, the POD activity of YT1 was enhanced by 11%, YT2 by 4%, and YT3 by 21% compared with YCK in response to drought stress (Fig. 5a). The H variety showed higher CAT activity (HT2 by 16%, HT3 by 39%, and HT1 had no statistical difference with HCK), while in Y cultivar reduction was observed in CAT activity (YT3 and YCK groups were found non-significant, 14 and 17% reduction for YT1 and YT2 in comparison with YCK, respectively) (Fig. 5b). In Fig. 5c, cultivar H exhibited improvement in APX activity by drought-hardening under drought stress. It had been observed that the APX activity of HT1 was enhanced by 26%, HT2 by 32%, and HT3 by 136% in comparison with control. Likewise, a 37% increment in the APX activity of YT3 was observed when subjected to drought stress in contrast to YCK. However, a 14% decline was observed in the APX activity of YT1 in comparison with the control. Finally, Fig. 5d exhibited that significant changes occurred in the activity of GR due to drought stress in the various drought-hardening treatments. The GR activity of the drought-hardened plants of HT1 and HT3 was increased by 19 and 30%, respectively, in comparison with control upon the drought stress imposition. Also, drought stress caused a gradual decline in the GR activity of the Y drought-hardened plants (YT1, YT2, and YT3) when compared with the non-drought-hardened plants (YCK). A total of 36, 31, and 18% drop was observed in the GR activity values of YT1, YT2, and YT3, respectively, compared with control.

### Changes in ascorbic acid (AsA), and glutathione (GSH) contents

**Fig. 6:**
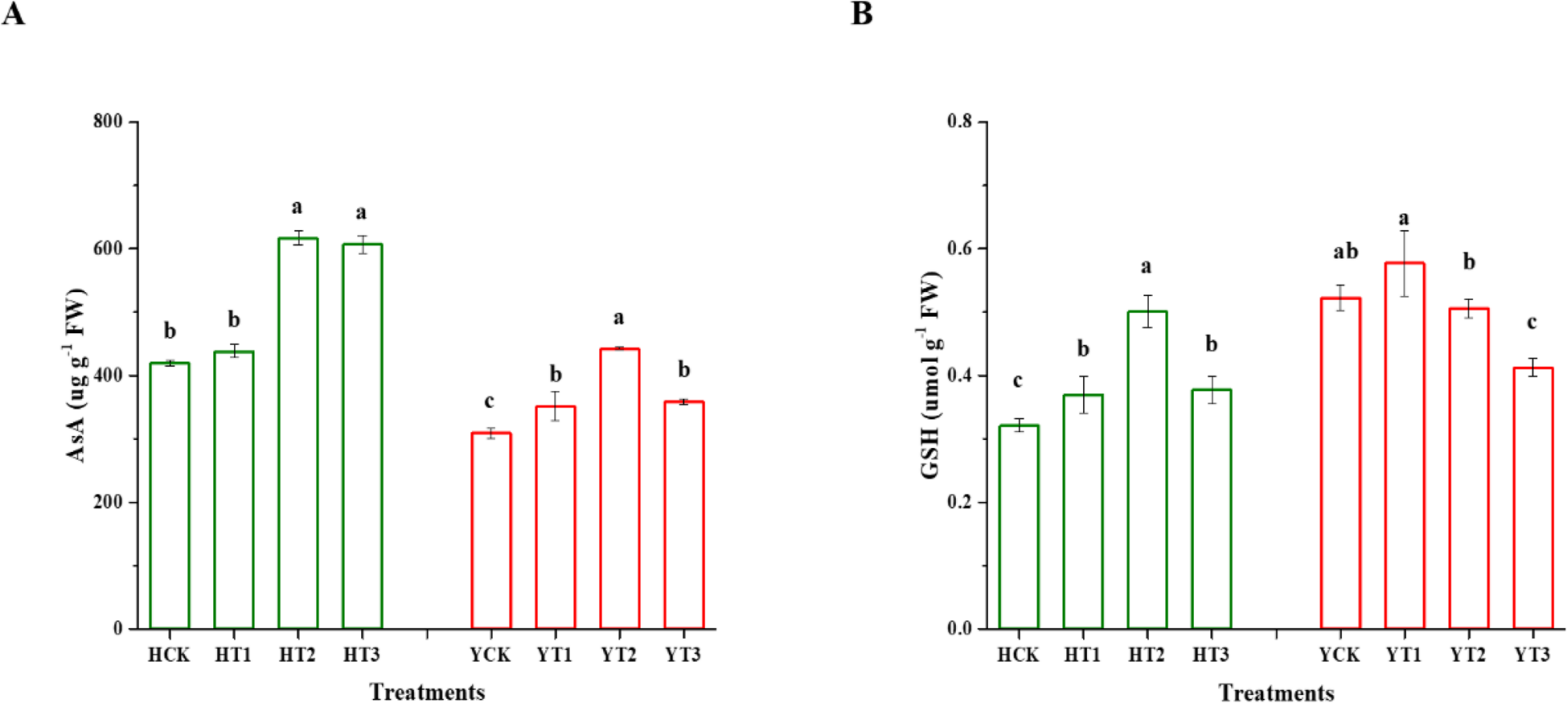
Effect of drought-hardening on the non-enzymatic antioxidant defense system in response to drought stress. Drought stress-induced changes in the contents of antioxidant substances, AsA (**a**) and GSH (**b**) of the two tobacco varieties of various drought-hardening treatments seedlings along with non-drought-hardened seedlings. The error bars on each mean value are ± SDs, different letters indicating significant differences at *p* < 0.05 by LSD test. HCK represents HongHuaDaJinYuan seedlings with no drought-hardening; HT1 indicates 24 h drought-hardened seedlings of HongHuaDaJinYuan; HT2 indicates 48 h drought-hardened seedlings of HongHuaDaJinYuan; HT3 indicates 72 h drought-hardened seedlings of HongHuaDaJinYuan; YCK represents Yun Yan-100 seedlings with no drought-hardening; YT1 indicates 24 h drought-hardened seedlings of Yun Yan-100; YT2 indicates 48 h drought-hardened seedlings of Yun Yan-100; YT3 indicates 72 h drought-hardened seedlings of Yun Yan-100

The AsA and GSH contents were significantly affected in the various drought-hardening treatments of both H and Y varieties under drought stress (Fig. 6). HT2 and HT3 augmented the AsA content by 47 and 45% in comparison with control, respectively. Figure 6a depicted that in the drought-hardened plants of YT1, YT2, and YT3, a significant rise of 14, 43, and 16% was observed in AsA contents in comparison with the non-drought-hardened plants (YCK) under drought stress. The results also showed a 56% increase of GSH content in drought-hardened plants of HT2 as compared to the non-drought-hardened plants (HCK) followed by HT3 and HT1 with 17 and 15% increment, respectively. Similarly, drought stress instigated a significant difference negatively in Y during different treatments. The drought-hardened plants of YT2 and YT3 exhibited a 3 and 21% reduction in GSH content in comparison with YCK in response to drought stress.

### Changes in proline and soluble sugar (SS) contents

**Fig. 7:**
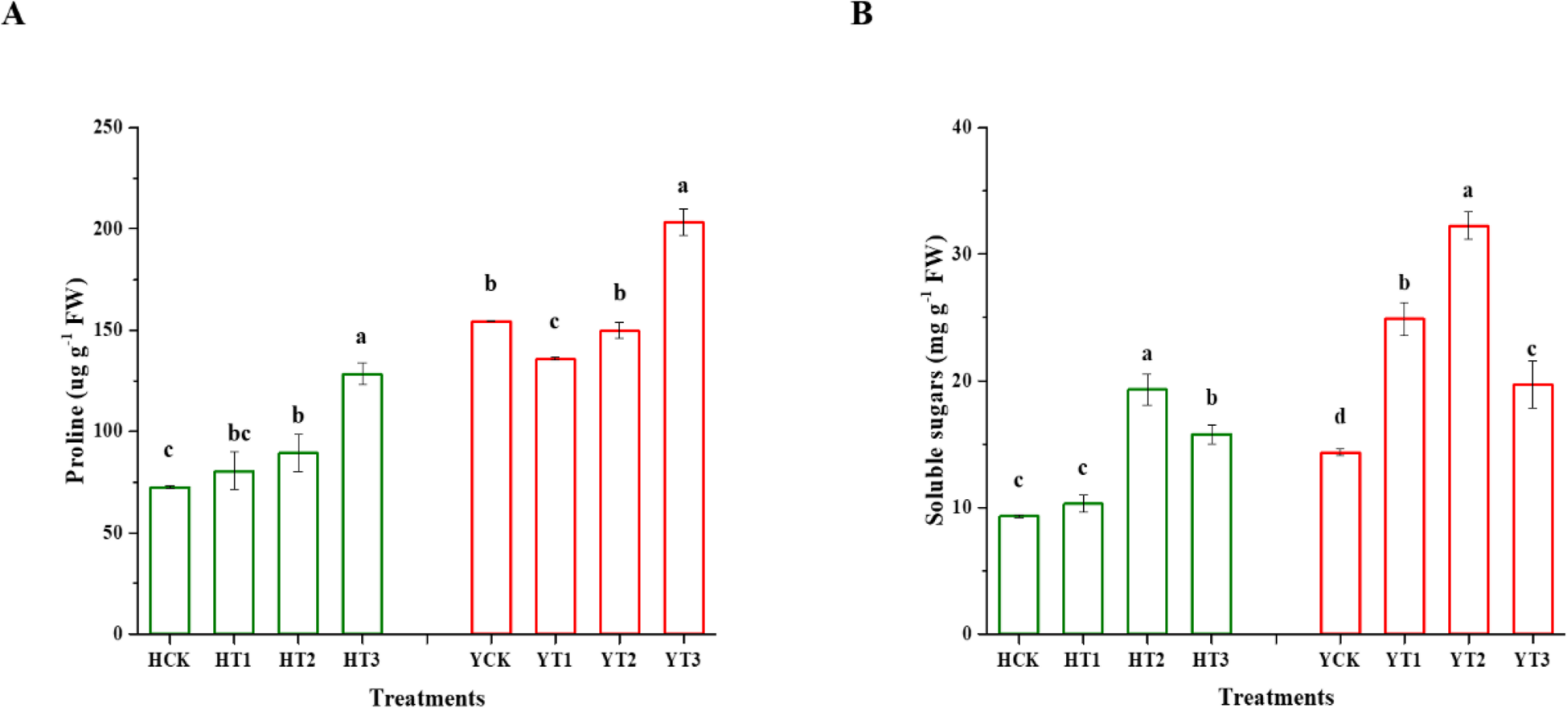
Effect of drought-hardening on proline and soluble sugar contents in response to drought stress. Drought-hardening enhanced the Proline (**a**) and Soluble Sugars (SS) (**b**) contents in the two tobacco variety seedlings under drought stress. The values are presented as means ± SDs, different letters indicating significant differences at *p* < 0.05 by LSD test. HCK represents HongHuaDaJinYuan seedlings with no drought-hardening; HT1 indicates 24 h drought-hardened seedlings of HongHuaDaJinYuan; HT2 indicates 48 h drought-hardened seedlings of HongHuaDaJinYuan; HT3 indicates 72 h drought-hardened seedlings of HongHuaDaJinYuan; YCK represents Yun Yan-100 seedlings with no drought-hardening; YT1 indicates 24 h drought-hardened seedlings of Yun Yan-100; YT2 indicates 48 h drought-hardened seedlings of Yun Yan-100; YT3 indicates 72 h drought-hardened seedlings of Yun Yan-100

Figure 7a portrayed the effect of drought-hardening on proline content in the two tobacco varieties in response to drought stress. Significant changes were observed in different treatments of both varieties in response to drought stress. An elevated amount of proline content with 77 and 23% rise was depicted in the drought-hardened plants of HT3 and HT2 in comparison with HCK. Drought stress caused a 32% rise in the proline content of YT3, and 12% reductions were witnessed in YT1 plants when compared with YCK. Similarly, under the drought stress, the soluble sugar content in HT2 and HT3 drought-hardened seedlings was 107 and 69% higher than those in non-drought-hardened plants, respectively. Besides, in response to drought stress, 74, 125, and 37% rise was observed in the seedlings of drought-hardened plants of YT1, YT2, and YT3 compared with YCK, respectively (Fig. 7b).

### Changes in leaf anatomical structure

**Fig. 8:**
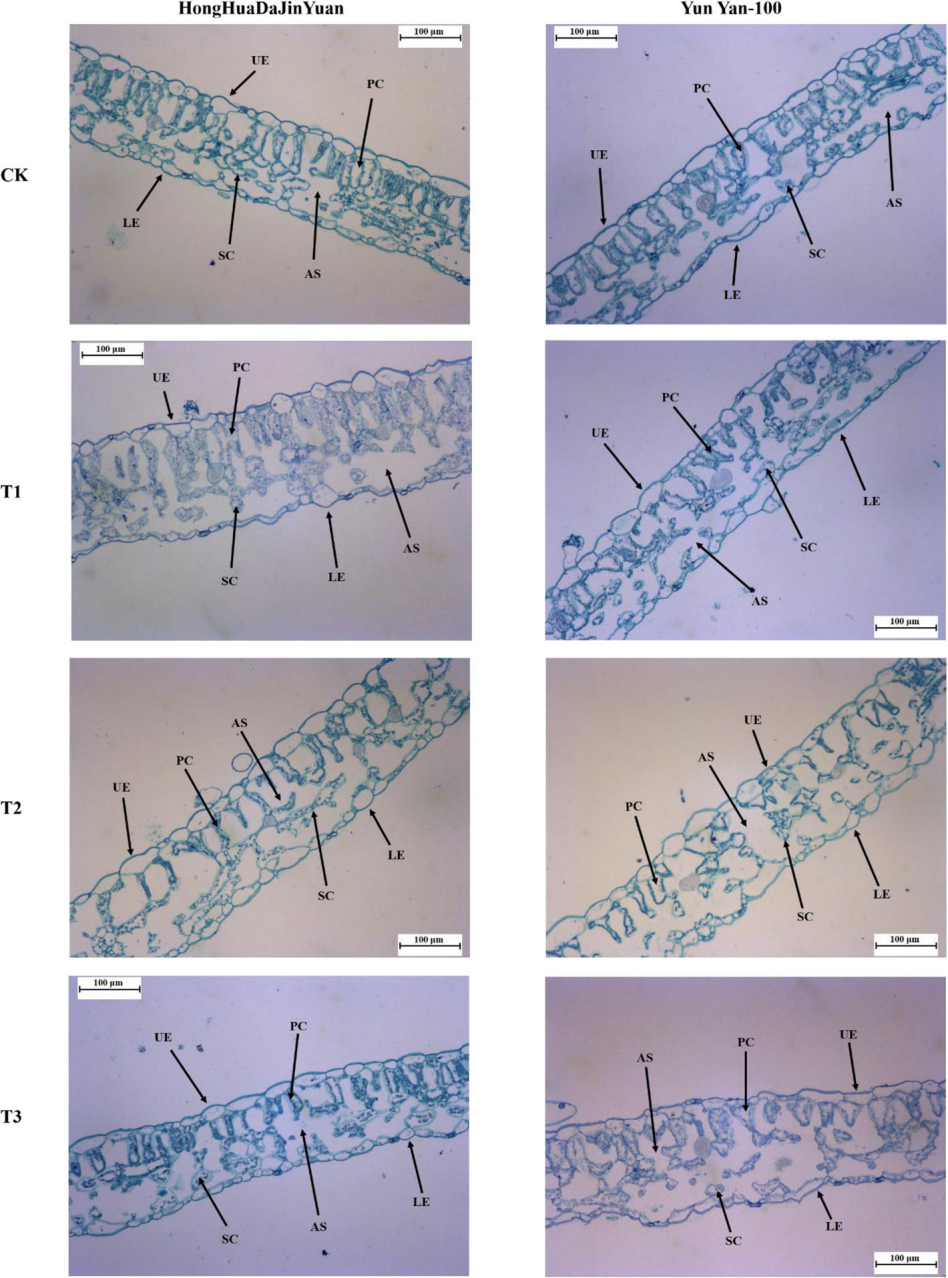
Effect of drought-hardening on the leaf anatomy of the two tobacco varieties under drought stress. Representative leaf anatomical images of the two tobacco varieties under drought stress of various drought-hardening treatments along with control. UE represents upper epidermis, LE represents lower epidermis, SC represents spongy cells, PC represents palisade cells, and AS represents air spaces. CK representing control/non-drought-hardened plants; T1 represents drought-hardening for 24 h; T2 represents drought-hardening for 48 h; T3 represents drought-hardening for 72 h

To study the leaf anatomical alterations, whether the drought-hardening had an impact on the leaf anatomy of the two varieties under the subsequent drought stress or not. The general structure of the leaves was maintained by application of drought-hardening under the subsequent drought stress conditions. It was still possible to distinguish the cells in the upper epidermis (UE), the lower epidermis (LE), palisade tissue (PC), and spongy tissue (SC) (Fig. 8). However, there is general disorganization of the cells in the overall leaf structure of both varieties. Especially, prominent disorganization, deformation, and larger intercellular spaces were observed in the palisade and spongy tissues in T2 and T3 (Fig. 8). It was also witnessed that the number of palisade cells was higher in T3 in comparison to other treatments.

### Transcriptional expression of antioxidant-, osmolyte biosynthesis-, and stress-related genes

**Fig. 9:**
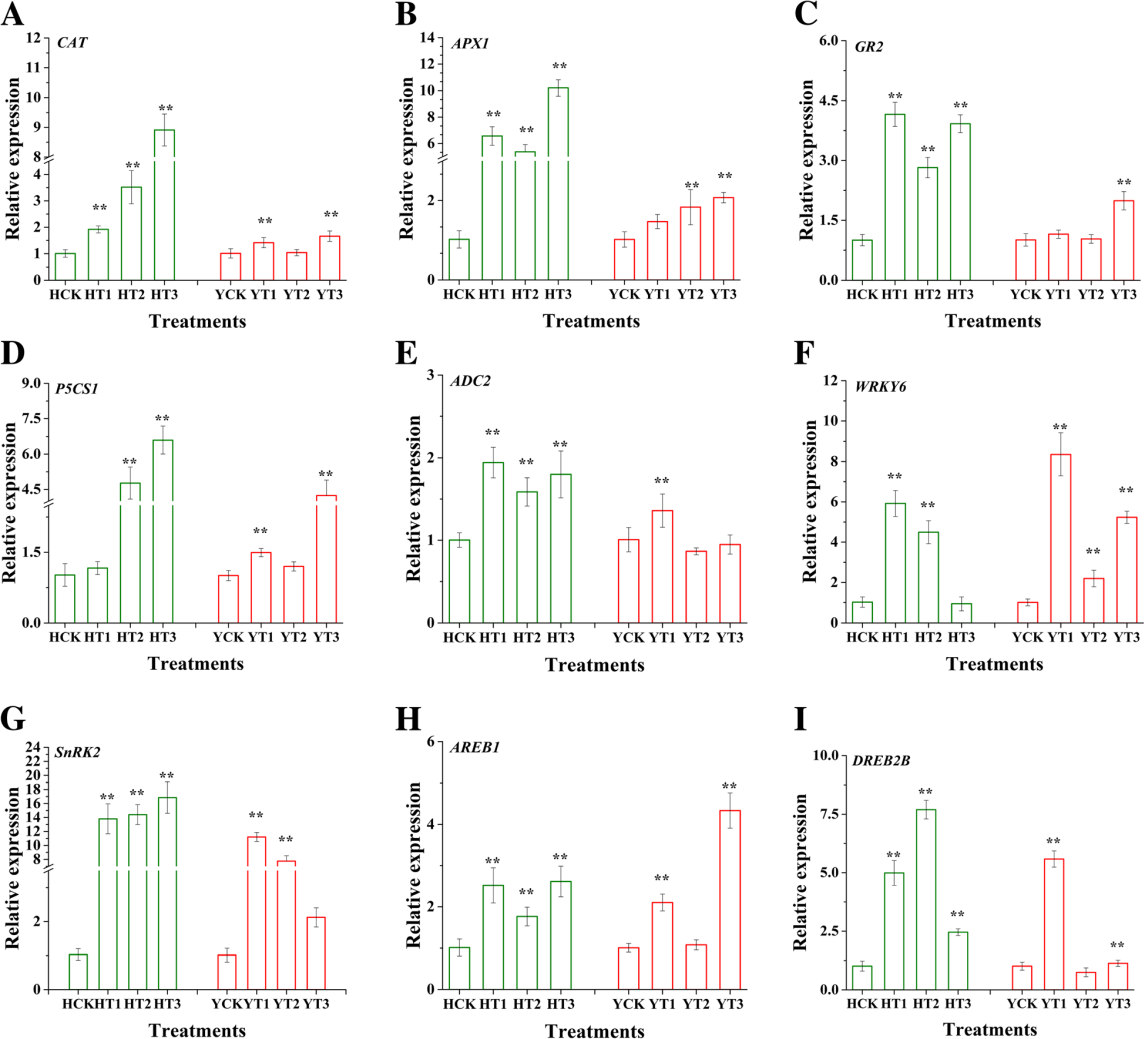
Effect of drought-hardening on various gene transcript levels in response to drought stress. Drought-hardening enhanced the expression of three antioxidant enzyme-related genes [*CAT* (**a**), *APX1* (**b**), and *GR2* (**c**)], two osmolyte biosynthesis-related genes [*P5CS1* (**d**) and *ADC2* (**e**)], one ABA signaling-related gene (*SnRK2*) (**g**), and three transcription factor-related genes [*AREB1* (**h**), *DREB2B* (**i**), and *WRKY6* (**f**)] in response to drought stress. HCK represents HongHuaDaJinYuan seedlings with no drought-hardening; HT1 indicates 24 h drought-hardened seedlings of HongHuaDaJinYuan; HT2 indicates 48 h drought-hardened seedlings of HongHuaDaJinYuan; HT3 indicates 72 h drought-hardened seedlings of HongHuaDaJinYuan; YCK represents Yun Yan-100 seedlings with no drought-hardening; YT1 indicates 24 h drought-hardened seedlings of Yun Yan-100; YT2 indicates 48 h drought-hardened seedlings of Yun Yan-100; YT3 indicates 72 h drought-hardened seedlings of Yun Yan-100

It has been well-reported that a number of genes are regulated by drought stress. In this study to put light on the regulatory mechanisms of two tobacco cultivars via drought-hardening against drought stress, the expression profiles of different categories genes were analyzed comprising of two osmolyte biosynthesis-related genes *P5CS1* and *ADC2*, three antioxidants-related genes *CAT*, *APX1*, and *GR2*, one gene related to ABA signaling *SnRK2*, and three transcription-related genes *AREB1*, *DREB2B*, and *WRKY6* (Fig. 9). The results demonstrated that the transcriptional levels of the three antioxidant defense system-related genes *CAT*, *APX1*, and *GR2* in both varieties were detected in drought-hardened treatment against drought stress. The expression levels of these genes were greatly increased in drought-hardened plants in comparison with control. It was also observed that the expression of these genes was more pronounced in T3 than other treatments (Fig. 9a-c). Similarly, the osmolyte biosynthesis-related genes *P5CS1* and *ADC2* also showed expression in drought-hardened plants in response to drought stress. The expression of *P5CS1* was more noticeable in T3 than other treatments in both cultivars. The expression levels of *ADC2* was elevated in H cultivar in drought-hardening treatments in comparison to control while its expression was only higher in YT1 (Fig. 9e). Finally, the expression of *SnRK2*, *AREB1*, *DREB2B*, and *WRKY6* genes were found significant in drought-hardening treatments in both cultivars (Fig. 9f-i). The expression of *WRKY6* was lower in HT3 than other treatments. In cultivar Y, its expression was increased by all the drought-hardening treatment in comparison with control in response to drought stress. Subsequently, drought-hardening treatments increased the expression levels of *SnRK2*, *AREB1,* and *DREB2B* genes against drought stress in both varieties.

## Discussion

It is a challenging task to better understand the drought tolerance at once; therefore, more research is needed at physiological, biochemical, and molecular levels to know the mechanisms underlying drought tolerance. Therefore, this research was planned for a better understanding of the drought-hardening effect on two tobacco cultivars during drought stress to inspect the drought tolerance and adaptation mechanisms. Plants were exposed to various drought-hardening treatments along with non-drought-hardened plants and then exposed to drought stress. In this study, drought-hardening improved the drought tolerance and adaptation of both varieties.

Drought is one of the major stresses among the other abiotic stresses which results in diminished crop growth and development. On one hand, drought-hardening treatments reduced the growth in both H and Y varieties in response to drought stress. The results of this study are in line with Makonya et al. [31] as witnessed lower shoot biomass allocation in chickpea due to drought priming. While, on the other hand, drought-hardening improved the water uptake ability. Leaf water potential is a reliable parameter which is used as an indicator for plant water status and as an index that reflects the physiological features of a plant drought tolerance [16]. Plants tissues with lower water potential can take up more water [32]. Thus our findings suggest that drought-hardening improved the water uptake ability, which is in line with Huang et al. [16].

### Manifestations in chlorophyll fluorescence and multicolor fluorescence parameters caused by drought-hardening in response to drought stress

The fundamental photosynthetic mechanisms in plants to various stresses can also be assessed via chlorophyll fluorescence techniques, which are the popular techniques used in plant physiology [33]. The QY_Lss is an important parameter which presents the efficiency of PSII photochemistry [34], qP_Lss represents the number of open PSII reaction centers [35], and Rfd_Lss is recognized for the photosynthetic capacity of plants also known as plants vitality [36]. In this study, drought-hardening negatively affected the qP_Lss, QY_Lss, and Rfd_Lss as a reduction was observed under the subsequent drought stress which resulted in a decrease in photosynthetic rates [35]. The results of this study were also coinciding with Wang et al. [37] in terms of reduction in actual photochemical efficiency by drought priming.

Chlorophyll fluorescence and MCF images (Fig. 3), both represent spatial heterogeneity. In chlorophyll fluorescence parameters, QY_Lss and qP_Lss, the intensity of the signals decreased at the tip and margins of the leaf, which showed a greater potential restriction in photosynthesis, while these variations were opposite in BF as the intensity of signals was higher at tip and margins of the leaf. It might be due to the breakdown of the chlorophyll into intermediary compounds and accumulation of phenolic compounds [38]. Thus, the phenolic compounds had a role in mitigating the adverse impacts of various abiotic stresses and helped in providing tolerance to multiple stresses [39].

### Drought-hardening alleviates the oxidative damage during drought stress

Oxidative stress has many biological targets, but lipids are the most targeted class of biomolecules [40]. Plants exposed to abiotic stresses engage in reactive oxygen species (ROS) production. The ROS attack membrane lipids in the cell cause lipid peroxidation and, consequently, cell death due to its cytotoxic role [41]. A well-known stress biomarker malondialdehyde (MDA) is generally used for the detection of the membrane lipid peroxidation in plants [40, 42]. Previous studies reported an elevated amount of MDA and H_2_O_2_ [43, 44] in response to drought stress indicated membrane damage. The findings of this study were supported by [19, 20] as they also witnessed lower MDA levels in drought-hardened plants. Similarly, in this study, drought-hardening treatments showed a differential response of H_2_O_2_ content in both varieties in response to drought stress. This difference in both varieties in terms of H_2_O_2_ contents might suggest that the two varieties had different response mechanism under same conditions and could provide drought tolerance in both varieties as it is a key molecule involved in various signaling pathways in conferring drought tolerance [45] via its involvement in stomatal closure [46].

### Drought-hardening imposes drought tolerance via enhanced enzymatic antioxidant defense system

Reactive oxygen species are the unavoidable substances generated both under normal and stress conditions, while they are toxic under stressful conditions. Various studies highlighted the role of antioxidant enzymes, and drought tolerance is correlated to the activities of these enzymes [47, 48]. It is well-known that to ensure survival and negate the adverse effects of ROS, plants activate the antioxidant enzymes defense system in response to an elevated amount of ROS [49, 50]. POD, CAT, APX, and GR are the main components of the enzymatic antioxidant defense system [51] which efficiently scavenge ROS and play an important role in conferring drought stress tolerance [51, 52]. CAT is the first line antioxidant enzyme [53], APX and GR are the key components of the Ascorbate-Glutathione (AsA-GSH) pathway which mitigates the harmful effects of ROS [54]. A previous study reported that drought-primed wheat plants activate their enzymatic antioxidant defense system to subsequent heat stress to alleviate the oxidative damage [55]. The findings of this study were supported by [19, 56] as drought-hardening improved the enzymatic plant defense system, thus conferring drought tolerance. In summary, the findings indicated that drought-hardened H and Y tobacco seedlings have a greater ability to reduce the oxidative damage caused by MDA and H_2_O_2_ thus maintain the stability of the cell membrane and helps in alleviating the damage induced by drought stress. It was concluded that drought-hardening is a fruitful strategy to provide drought tolerance and adaptation in tobacco seedlings via enhancing the plants’ antioxidant defense system.

### The positive impact of drought-hardening on non-enzymatic antioxidant defense system results in drought tolerance

The non-enzymatic antioxidant defense system plays multiple roles in cellular defense throughout the plant life cycle. Ascorbic acid (AsA) plays multiple roles in plant growth and development under both stress and non-stress conditions. It helps in maintaining the low levels of ROS in plants under stressful conditions as it is considered as a universal non-enzymatic antioxidant [57]. Extreme adverse environmental conditions aggravate ROS production, which alters the intracellular redox environment; thus, GSH keeps cellular redox poise, protects proteins from denaturation, and helps in chelating of toxic metals, which triggers adaptive responses [58]. It is also considered as the most powerful non-enzymatic antioxidant substance by quenching ROS species [59]. AsA and GSH, the non-enzymatic antioxidant substances, play a role in either preventing or in lowering the danger caused by ROS in crop plants. Previous studies documented the role of AsA and GSH in ROS scavenging in wheat [60] and potato [61]. A previous study had shown that drought priming induced the non-antioxidant defense system (AsA and GSH contents) to mitigate the harmful effects of ROS [62]. Drought-hardening resulted in an elevated amount of both AsA and GSH contents mitigating the negative effects of ROS thus suggesting that drought-hardening play a role in drought-tolerance and adaptation in response to drought stress and it is well established that both AsA and GSH play a role in conferring drought tolerance in plants [57, 58].

### Drought-hardening increases the content of proline and soluble sugars involved in mitigating the adverse effects of drought stress

Proline and soluble sugars (SS) are involved in performing vital roles in plants under stress conditions. Both act as osmoprotectants, signaling molecule, keeps redox balance in cell, scavenge the free radicals, stabilize cellular structures, and eventually assists the plants to recover from stressful conditions [63, 64]. Proline accumulation is a vital osmotic regulator in plants and plays a key role in the maintenance of turgor pressure and maintain osmotic balance, which is an adaptive mechanism involved in drought tolerance [65, 66]. Soluble sugars are also involved in playing a dual role in plants under stress conditions, as a signaling molecule and osmoregulatory substance [64]. Various studies showed that proline and soluble sugars are involved in drought tolerance and adaptation in many plant species [67–70]. Proline and SS contents showed a differential response to the duration of drought-hardening treatments. Proline contents increased gradually with an increase in drought-hardening time while SS contents reached to its peak at 48 h of drought-hardening against subsequent drought stress. The findings of this study pointed out an increase in the proline and SS contents in drought-hardened plants of both varieties under drought stress, which were in line with Zhang et al. [19] and Yang et al. [20]. Osmoregulation is a key factor and an important strategy of plants to provide drought tolerance; proline and soluble sugars are the key substances involved in the osmoregulation process [71]; thus the elevated amounts of proline and SS due to drought-hardening resulted in osmoregulation in the two tobacco varieties as also witnessed in Jatropha drought-hardened seedlings and thus enhances drought tolerance [20].

### Leaf anatomical modifications of drought-hardened and non-drought-hardened seedlings under drought stress

Plants make several changes in their leaf anatomy to ameliorate the effects of drought stress. Mesophyll cells are comprised of two layers, palisade cells and spongy cells; palisade cells help in the process of photosynthesis, give mechanical support, and prevent water loss, while spongy tissues store a lot of water and help in gaseous exchanges [19]. Cell size was reduced in response to drought stress [72]. Small cells can be helpful under water stress condition and lower mesophyll cell density was observed as in previous study [73] which may contribute to drought tolerance, as plants or tissues with reduced cell size are more resistant to water stress [74]. Drought-hardening resulted in a decrease of palisade cells and their number in both varieties under subsequent drought stress. The results are in line with Zhang et al. [19] as drought-hardening developed-well palisade tissue in potato.

### Drought-hardening improves the expression of potential drought-responsive genes in response to drought stress

In the present study, the expression profile of antioxidant-related genes, *CAT*, *APX1*, and *GR2,* showed upregulation in drought-hardening treatments in both cultivars in response to drought stress. Previous studies demonstrated that *CAT*, *APX,* and *GR* gene expression were upregulated in response to drought stress, thus confer stress tolerance [75, 76]. The upregulation of *CAT* and *APX1* genes is consistent with the enzyme activities in both varieties. Drought-primed wheat plants showed higher expression levels of *APX1* [62] which support the results of this study. The expression of the *GR2* gene and GR enzyme activity was not in accordance with each other in both varieties in the various drought-hardening treatments. The presence of its different isoforms may explain this. The assayed enzyme activity has resulted in more than one isoform activity, while in this study, only one isoform expression level was tested. This contradiction between the enzyme activity and its gene expression can be elucidated as by isogene specificity [25], and gene expression cannot be directly correlated with the enzyme activity because of the complex regulatory mechanisms of gene expression [24].

Proline accumulation in plants under stressful conditions contributed to stress tolerance as it plays multiple roles under stress conditions [77]. Dudzaik et al. [78] reported higher expression of *P5CS* gene in wheat under drought stress. *P5CS1* is a key gene involved in the biosynthesis of proline under stress conditions [79] its activity was enhanced by drought-hardening treatments in both H and Y cultivars under drought stress and showed consistency with the assayed proline content. The findings of this research were supported by [20, 62] as drought-hardening improved both the proline content and expression of *P5CS1* gene. Similarly, *ADC2* regulates the biosynthesis of the polyamine, and polyamines are involved in stress tolerance under various abiotic stresses [80, 81]. Drought-hardening treatments resulted in enhanced expression of the *ADC2* gene. In conclusion, drought-hardening conferred drought tolerance in response to drought stress, which was partly due to the activation of proline and polyamines biosynthesis pathways.

*SnRK2* (involved in ABA signal transduction), *AREB1*, *DREB2B*, and *WRKY6* are important transcription factors that might induce stress-related genes and thus confer stress tolerance. Different genetic approaches and modulation of these transcription factors are involved in various abiotic stress tolerance and adaptations [82, 83]. Previous researches highlighted the role of *SnRK2*, *AREB*, *DREB,* and *WRKY* genes in drought tolerance and adaptation in grape [30], wheatgrass [84], and soybean [85]. Yoshida et al. [86] demonstrated that *AREB* is downstream stress-responsive transcription factors of *SnRK2* in response to osmotic stress. The findings of this study revealed that overall, various drought-hardening treatments triggered both ABA-dependent and independent pathways that might regulate the transcriptional networks in response to drought stress in H and Y varieties, which resulted in drought tolerance. Similarly, heat tolerance of *Arabidopsis* and tall fescue was associated with drought priming by the upregulation of *AREB* and *DREB* genes [87]. Other studies also found the regulation of ABA-dependent and independent pathways in response to drought stress [28, 30]. Overall, in this study, the data showed differential expression of the analyzed genes in various drought-hardening treatments of the two varieties under drought stress, which proposes that each variety utilizes different molecular responses under the same conditions [24].

## Conclusions

In conclusion, taken together, the results of this study provide a complete framework of drought-hardening effect at physiological, biochemical, and gene expression levels with which two tobacco varieties were given various drought-hardening treatments along with control in response to drought stress. Overall, the drought-hardening improved the drought tolerance and adaptation of both varieties. Drought-hardening treatments, especially T2 and T3, improved both the enzymatic and non-enzymatic antioxidant defense systems to mitigate the negative impacts of oxidative damage. T3 and T2 accumulated higher levels of proline and SS in both varieties, respectively, and thus helped in bringing osmoregulation. Finally, the drought-hardening induced drought tolerance was partly due to the expression of various stress-responsive genes by triggering the biosynthesis pathways of proline, polyamines, ABA-dependent and independent pathways, and antioxidant defense-related genes (*CAT*, *APX1*, and *GR2*) in response to drought stress.

## Methods

### Plant materials, growth conditions, and drought-hardening

**Fig. 10:**
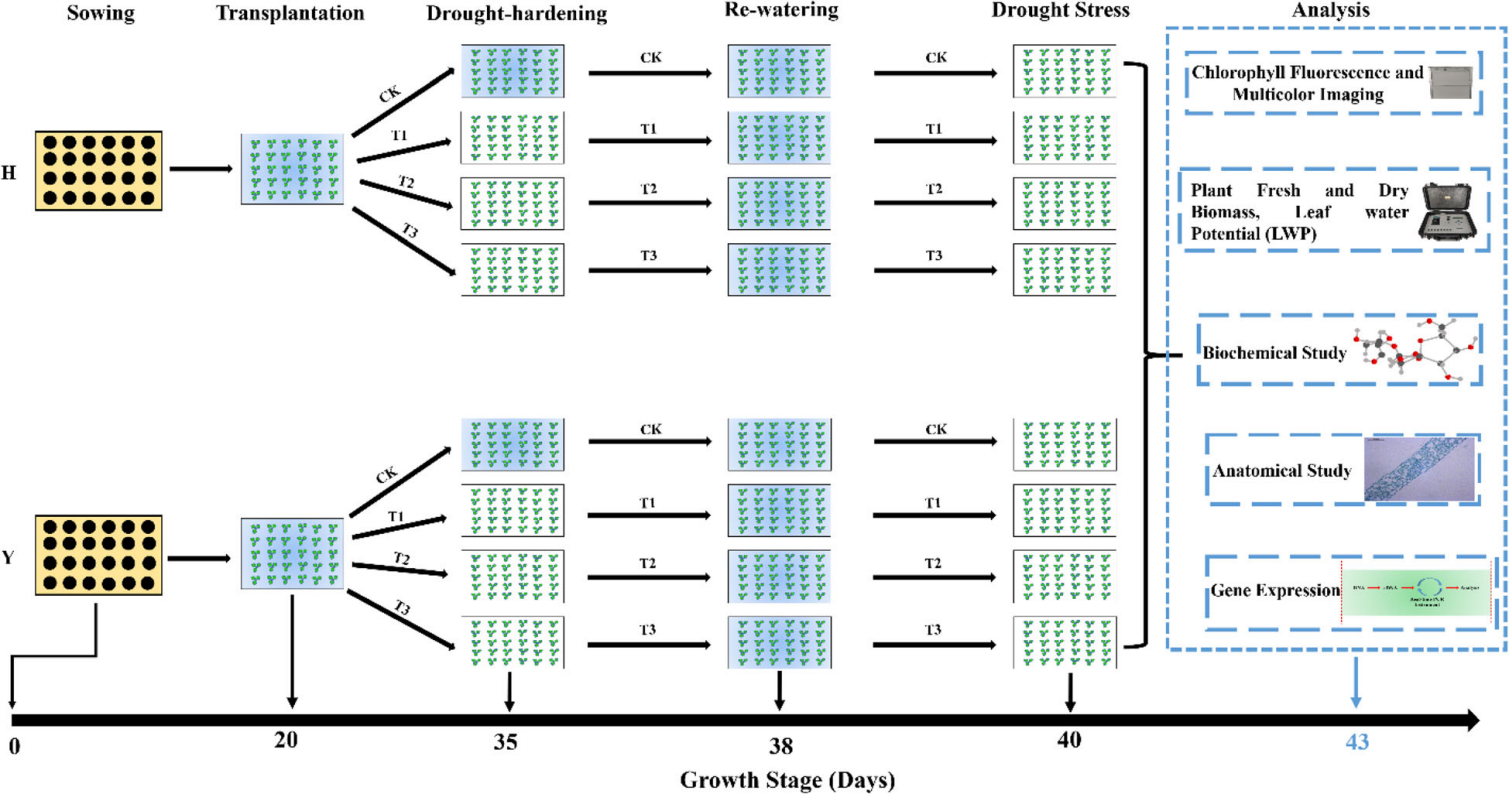
This schematic flow shows the setup of the experiment. Drought-hardening was applied to two tobacco variety seedlings and then subjected to drought stress. H and Y representing HongHuaDaJinYuan and Yun Yan-100, respectively. CK representing control/non-drought-hardened plants; T1 represents drought-hardening for 24 h; T2 represents drought-hardening for 48 h; T3 represents drought-hardening for 72 h. Trays with blue color present normal water conditions, while trays with white color present seedlings under drought stress conditions

This study was designed to evaluate the drought tolerance through physiological, biochemical, and expression of various gene transcripts analyses by applying drought-hardening to two *Nicotiana tabacum* varieties, namely HongHuaDaJinYuan (H) and Yun Yan-100 (Y). HongHuaDaJinYuan and Yun Yan-100 were selected by the Yunnan Academy of Tobacco Agriculture Science. After the DUS (Distinctness, Uniformity, and Stability) test and certification from the National Tobacco Variety Certification Committee, HongHuaDaJinYuan and Yun Yan-100 were kept by the National Infrastructure for Crop Germplasm Resource Tobacco (Qingdao, China) with a national serial number’s “00000540” and “00004851”, respectively. This research was carried out in the Tobacco Research Institute of the Chinese Academy of Agricultural Sciences, Qingdao, China. The experiment was performed in control conditions using the floating breeding system. The floating breeding is a system in which the potted seedlings or seedlings sown in white foam floating trays are placed into water or nutrient solutions [88]. The plants were grown at 26 °C with 300 μmol m^− 2^ s^− 1^ of light intensity. The relative humidity was 45% and 12 h lengths of light and dark periods. Plants of the uniform size were selected for the experiment. Drought-hardening was applied at different levels to two tobacco variety seedlings by withdrawing water (water-break) from the floating breeding system. The treatment plants were divided into four groups, CK (no drought-hardening), T1 (drought-hardening for 24 h), T2 (drought-hardening for 48 h), and T3 (drought-hardening for 72 h). The plants were re-watered for 48 h after applying drought-hardening. In the end, again, drought stress was given for 72 h. Samples were taken from different treated plants and frozen immediately in liquid nitrogen. The frozen samples were then stored at -80 °C for further analysis. The sketch of the entire experiment is presented in Fig. 10.

### Measurement of plants fresh and dry biomass, and leaf water potential (LWP)

The plant samples were taken and immediately their fresh weight was recorded. After measuring the fresh weight of the plants, all the plant samples were then dried in an oven at 80 °C for 48 h and weighed.

The young fully expanded similar size leaf of the same position from the two tobacco cultivar of CK, T1, T2, and T3 seedlings were chosen for finding LWP. The Ψ_leaf_ as MPa was recorded by keeping the circular leaf disks using the PSYPRO water potential system (WESCOR, Logan, UT, USA) in a sealed C-52 psychrometer chamber [89].

### Determination of chlorophyll and multicolor fluorescence imaging

Pulse amplitude modulated chlorophyll fluorescence imaging system (FluorCam FC 800, Photon Systems Instruments, Brno, Czech Republic) was used at room temperature in the lab of Tobacco Research Institute, CAAS, Qingdao, China. The leaf chlorophyll fluorescence parameters measurement was carried out on a second fully expanded detached leaves from the apex of the plants. The leaves were kept for 20 min in the dark which allowed the leaves to open its PSII reaction centers. The leaf was kept under the FluorCam and Fo was measured. After Fo, Fm was measured with a pulse duration of 800 ms. Then the leaf was relaxed in dark for 17 s. The Fm quenching analysis was carried out on the leaf for 70 s. In the 70 s duration time, Fp was measured after 2 s, Fm_L1 was measured after 8 s, Fm_L2, Fm_L3, Fm_L4, and Fm_Lss were measured after 18 s, 28 s, 48 s, and 68 s, respectively, after its exposure to a constant 100 μmol m^− 2^ s^− 1^ actinic light. After the actinic light period, the leaf was again dark-relaxed for further 100 s to measure Fm_D1 (28 s), Fm_D2 (58 s), and Fm_D3 (88 s). Based on these basic chlorophyll fluorescence signals the steady-state PSII quantum yield (ΦPSII_Lss), qP_Lss (open reaction centers), and Rfd_Lss (ratio of fluorescence decline) can be derived as equations [90].

$$ \Phi \mathrm{PSII}\_\mathrm{Lss}=\left(\mathrm{Fm}\_\mathrm{Lss}-\mathrm{Ft}\_\mathrm{Lss}\right)/\mathrm{Fm}\_\mathrm{Lss} $$

$$ \mathrm{qP}\_\mathrm{Lss}=\left(\mathrm{Fm}\_\mathrm{Lss}-\mathrm{Ft}\_\mathrm{Lss}\right)/\left(\mathrm{Fm}\_\mathrm{Lss}-\mathrm{Fo}\_\mathrm{Lss}\right) $$

$$ \mathrm{Rfd}\_\mathrm{Lss}=\left(\mathrm{Fp}-\mathrm{Ft}\_\mathrm{Lss}\right)/\mathrm{Fm}\_\mathrm{Lss} $$

Similarly, an ultraviolet (UV) (320–400 nm) LED panel as an excitation source was used for multicolor fluorescence imaging (MFI). The leaves from the various treatments of drought-hardened seedlings after applying drought stress were exposed to the UV light. Finally, the blue fluorescence (BF) data and its images were obtained from the whole MFI data set [90].

Note: The raw data used for plant growth, leaf water potential, chlorophyll fluorescence, and multicolor fluorescence parameters were presented in Data Set 1 (supplementary data).

### Measurement of biochemical parameters

Leaf samples were collected in liquid nitrogen from the two tobacco varieties of drought-hardening treatments and control plants after applying drought stress. The leaf samples were then stored at -80 °C for further analysis. Reactive oxygen species (H_2_O_2_, MDA), antioxidant enzyme (POD, CAT, APX, GR) activities, non-enzymatic antioxidant substance (AsA, GSH) contents, and osmoregulation substance (proline, soluble sugars) contents were determined spectrophotometrically by SHIMADZU UV-2600 spectrophotometer (Shimadzu Corporation, Kyoto, Japan) using the commercial kits as per the manufacturer guidelines. The commercial kits were purchased from Suzhou Comin Biotechnology Co., Ltd., Jiangsu, China.

Note: Please see the Online Resource 1 file in which the reagents details are listed used in biochemical analysis. Please also see the Data Set 2 (supplementary data) which presented the raw data used for the biochemical analysis.

### Quantification of antioxidant enzymes

The activity of POD was measured after grounding 0.1 g leaf samples in 1 mL of extraction solution and then centrifuged for ten minutes with 8000 rpm at 4 °C. Take 50 μL sample supernatant and 950 μL working solution and thoroughly mixed it. After mixing, the absorbance was recorded at 470 nm. The working solution was made from thoroughly mixing of the reagent 1, reagent 2, and reagent 3 of the commercial kit. One unit of the POD was the change in the OD absorbance at 2 min and 1 min [91].

The decomposition of H_2_O_2_ determined the CAT activity [91] by reading the absorbance at 240 nm for one min after the grounding and centrifugation of leaf samples in 1 mL of extraction solution at 8000 rpm for 10 min at 4 °C.

The APX activity was assayed spectrophotometrically. It was carried out by taking 0.1 g leaf samples and ground in 1 mL of extraction solution. After grounding leaf samples, the homogenates were centrifuged at 4 °C for 20 min with 13,000 rpm. After collecting the supernatant, we took 50 μL sample supernatant, 700 μL reagent 1, 100 μL reagent 2 and 100 μL reagent 3 of the commercial kit and mixed it thoroughly. After mixing, the OD at 290 nm for 1 min was recorded. One unit of the APX activity was defined as the amount of enzyme required to catalyzed 1 μmol ascorbate at 290 nm [92, 93].

The activity of GR was assayed by the grounding of 0.1 g leaf samples in 1 mL of extraction solution. After grounding the samples, the homogenates were centrifuged for 15 min with 8000 rpm at 4 °C. After centrifuge, the supernatant was collected. By taking 100 μL sample supernatant, 750 μL reagent 1, 100 μL reagent 2, and 50 μL reagent 3, mixed it quickly and immediately read the absorbance at 340 nm. One unit of GR activity was defined as the amount of enzyme that oxidized 1 μmol NADPH min^− 1^ [92, 93].

### Quantification of non-enzymatic antioxidant substances

Briefly, leaves (0.1 g) of the various treatments were homogenized in 1 mL of extraction solution. The centrifugation of the sample containing solution was carried out at 4 °C for 20 min using 8000 rpm. The supernatant of the samples was collected. 200 μL sample supernatant was taken and 60 μL reagent 1, 100 μL reagent 2, 240 μL reagent 3, and 1400 μL dd H_2_O was added and mixed equally for the quantification of ASA. The absorbance was recorded at 420 nm [94].

The reduced glutathione (GSH) was determined by weighing 0.1 g leaf samples and ground in 1 mL of extraction solution. After grinding, the homogenized solution was centrifuged for 10 min at 4 °C with 8000 rpm. Subsequently, 100 μL sample supernatant was taken and evenly mixed with 700 μL reagent 1 and 200 μL reagent 3. The OD was measured at 412 [94].

### Quantification of proline and soluble sugar contents

The proline levels were quantified as 0.1 g leaves were homogenized in 1 mL of the extraction solution. The homogenate was placed in a water bath for 10 min at 95 °C and then centrifuged at 10000 rpm for 10 min at 4 °C. 500 μL sample supernatant was taken and 500 μL reagent 1 (acetic acid) and 500 μL reagent 2 were added and thoroughly mixed. After mixing, again put it in the water bath for 30 min at 95 °C. Then cool the solution to room temperature and finally add 1 mL reagent 3 (toluene). The absorbance of the reaction mixture was read at 520 nm [95].

Soluble sugars were quantified in leaf samples ground with 1 mL of the extraction solution and then kept it in the water bath at 95 °C for 10 min. Then the samples were centrifuged at 25 °C for 10 min with 8000 rpm. The supernatant was collected, 40 μL ddH_2_O, 20 μL reagent 2, 200 μL reagent 3 (conc. Sulfuric acid) was added, and kept the solution mixture in an ice bath for 10 min at 95 °C. The absorbance of the supernatant was determined at 620 nm [95].

### Assay of malondialdehyde (MDA) and hydrogen peroxide (H_2_O_2_)

In summary, the MDA content in a 0.1 g sample was determined in a 1 mL homogenized extraction solution and centrifuged for 10 min at 4 °C with 8000 rpm. In the 0.2 mL sample supernatant, a 0.6 mL reagent 1 was added and kept it in a water bath at 95 °C for 30 min. Again the supernatant was centrifuged using 10,000 rpm for 10 min at 25 °C. The absorbance of the supernatant was read at 532 and 600 nm [96].

The H_2_O_2_ content was assayed by grounding the leaves with an addition of 1 mL extraction solution (acetone) and then centrifuged using 8000 rpm at 4 °C for 10 min. After collecting the supernatant, 100 μL reagent 2 and 200 μL reagent were added and evenly mixed. Then centrifugation of the solution mixture was carried out at 4000 rpm for 10 min at 25 °C. After centrifuge, the supernatant was discarded and sediments remained in the tube. Then we added 1 mL reagent 4. The absorbance of the reaction mixture was monitored spectrophotometrically at 415 nm [91].

### Anatomical studies

The anatomical analysis was performed on the samples taken from the mid-portion between the midrib and margin of the leaf from CK, T1, T2, and T3 treatments of the two drought-stressed tobacco varieties. After excising and collecting the samples, these were fixed in 70% FAA (formalin: acetic acid: alcohol) solution. The samples were dehydrated in different concentrations of ethanol (70–100%) and also processed with different xylene solutions. The samples were embedded in paraffin (60 °C) [97]. Thin slices from the leaf samples were formed and kept on the glass slides. The glass slide having the specimens were rehydrated and cleared using a series of xylene and ethanol solutions and then stained with Safranin O and fast green dyes and then mounted. Finally, the observations were performed on the slides using a microscope (Leica DM 2000, Wetzlar, Germany) and photographed with a Leica DMC 2900 camera (Leica, Wetzlar, Germany).

### RNA extraction, cDNA synthesis, and qRT-PCR

The total RNA was extracted from three biological repeats of drought-hardened and non-drought-hardened tobacco leaves of H and Y varieties using MiniBEST Plant RNA Extraction Kit (TaKaRa, Japan), according to the manufacturer instructions. The concentration of RNA was determined by Nanophotometer P330 (Implen, Munich, Germany) and its quality was assessed by agarose gel electrophoresis. After the extraction of RNA, the cDNA was synthesized via the PrimeScript 1st Strand cDNA Synthesis kit (TaKaRa, Japan), following the manufacturer protocol. The qRT-PCR was subsequently performed on Applied Biosystems QuantStudio3 real-time PCR machine (Applied Biosystems) on a total reaction volume of 20 μL using SYBER Green Master Mix (TaKaRa, Shiga, Japan). The qRT-PCR data were analyzed using the 2^-△△CT^ method [98]. The gene-specific primers (Online Resource 2) were designed using PRIMER3 (https://www.ncbi.nlm.nih.gov/tools/primer-blast/). The *Actin* was used as a reference internal control gene. The raw data for qRT-PCR analysis was presented in Data Set 3 (supplementary data).

### Statistical analysis

The data was statistically analyzed using One-way ANOVA in Statistix 8.1 (Analytical Software, Tallahassee, FL, USA). The differences in the mean values of the two varieties between the drought-hardening and control-treated plants were presented as the mean ± SD (Standard deviation) of the three replicates. The significant variations in the mean values are shown at *p* < 0.05 or *p* < 0.01 levels using LSD (least significant difference) test. The graphs were plotted using OriginPro 9.1 (OriginLab Corporation, Northampton, MA, USA).

## Supplementary information

**Additional file 1 **: **Online Resource 1**. Reagents list. **Online Resource 2**. Genes-specific primers list for qRT-PCR. **Data Set 1**. Raw data for plant growth, leaf water potential, chlorophyll fluorescence, and multicolor fluorescence analysis. **Data Set 2**. Raw data for biochemical analysis. **Data Set 3**. Raw data for qRT-PCR analysis.

## Data Availability

All data generated or analyzed during this study are included in this published article (and its supplementary information files).

## Abbreviations

APX: Ascorbate peroxidase
AsA: Ascorbic acid
BF: Blue fluorescence
CAT: Catalase
cDNA: Complementary DNA
CK: Control or no drought-hardening
GF: Green fluorescence
GSH: Reduced glutathione
H: HongHuaDaJinYuan
POD: Peroxidase
qRT-PCR: Quantitative real-time polymerase chain reaction
ROS: Reactive oxygen species
SOD: Superoxide dismutase
T1: Drought-hardening for 24 h
T2: Drought-hardening for 48 h
T3: Drought-hardening for 72 h
Y: Yun Yan-100
